# In-context learning enables large language models to achieve human-level performance in spinal instability neoplastic score classification from synthetic CT and MRI reports

**DOI:** 10.1007/s11547-025-02096-7

**Published:** 2025-09-24

**Authors:** Maximilian F. Russe, Marco Reisert, Anna Fink, Marc Hohenhaus, Julia M. Nakagawa, Caroline Wilpert, Carl P. Simon, Elmar Kotter, Horst Urbach, Alexander Rau

**Affiliations:** 1 Department of Diagnostic and Interventional Radiology, Faculty of Medicine, Medical Center – University of Freiburg, University of Freiburg, 79106 Freiburg, Germany; 2 Medical Physics, Department of Diagnostic and Interventional Radiology, Faculty of Medicine, Medical Center – University of Freiburg, University of Freiburg, 79106 Freiburg, Germany; 3 Department of Stereotactic and Functional Neurosurgery, Faculty of Medicine, Medical Center – University of Freiburg, University of Freiburg, 79106 Freiburg, Germany; 4 Department of Neurosurgery, Faculty of Medicine, Medical Center – University of Freiburg, University of Freiburg, 79106 Freiburg, Germany; 5 Department of Neuroradiology, Faculty of Medicine, Medical Center – University of Freiburg,, University of Freiburg, Hugstetter Str. 55, 79106 Freiburg, Germany

**Keywords:** Spinal metastasis, Large language model, SINS classification, In-context learning

## Abstract

**Purpose:**

To assess the performance of state-of-the-art large language models in classifying vertebral metastasis stability using the Spinal Instability Neoplastic Score (SINS) compared to human experts, and to evaluate the impact of task-specific refinement including in-context learning on their performance.

**Material and methods:**

This retrospective study analyzed 100 synthetic CT and MRI reports encompassing a broad range of SINS scores. Four human experts (two radiologists and two neurosurgeons) and four large language models (Mistral, Claude, GPT-4 turbo, and GPT-4o) evaluated the reports. Large language models were tested in both generic form and with task-specific refinement. Performance was assessed based on correct SINS category assignment and attributed SINS points.

**Results:**

Human experts demonstrated high median performance in SINS classification (98.5% correct) and points calculation (92% correct), with a median point offset of 0 [0–0]. Generic large language models performed poorly with 26–63% correct category and 4–15% correct SINS points allocation. In-context learning significantly improved chatbot performance to near-human levels (96–98/100 correct for classification, 86–95/100 for scoring, no significant difference to human experts). Refined large language models performed 71–85% better in SINS points allocation.

**Conclusion:**

In-context learning enables state-of-the-art large language models to perform at near-human expert levels in SINS classification, offering potential for automating vertebral metastasis stability assessment. The poor performance of generic large language models highlights the importance of task-specific refinement in medical applications of artificial intelligence.

## Introduction

Spinal metastases pose significant challenges in oncological care, affecting up to 40% of patients with advanced malignant disease. They are associated with severe pain, reduced mobility, neurological deficits, and spinal instability [1, 2]. Here, imaging is crucial for the identification of spinal metastasis. Additionally, it allows for identifying constellations that are prone to impair spinal stability and thus require distinct treatment strategies. For this, the Spinal Instability Neoplastic Score (SINS) has been established as a standard tool for evaluating instability in neoplastic spinal disease [3, 4], with clinicians and radiologists generally performing well in attributing the relevant categories (i.e., stable, potentially unstable, or unstable) [5]. The SINS classifies vertebral metastasis as either stable, potentially unstable, or unstable. The attribution relies on a scoring system that employs imaging features to evaluate the location, appearance, spinal alignment, presence of vertebral body collapse, and involvement of the posterior elements as well as the presence of pain.

Patients classified as stable (0–6 points) are at minimal risk of spinal instability or acute neurological compromise. Non-surgical management is typically sufficient in these cases, and treatment primarily relies on focuses on systemic therapies to address the underlying malignancy. In the potentially unstable category (7–12 points), patients are at intermediate risk of instability, which might potentially require treatment like an unstable lesion. Patients classified as unstable (13–18 points) are at high risk of spinal instability or acute neurological deterioration, and surgical intervention is often required to stabilize the spine. This includes decompression, fixation, or vertebral column reconstruction. This is frequently combined with radiotherapy, which can be administered alone, too.

The SINS is especially valuable as the category has direct clinical implications and clinicians and radiologists generally perform well in attributing the relevant categories (i.e., stable, potentially unstable, or unstable [5].

Nevertheless, studies have revealed substantial variability in precise SINS interpretation among clinicians [5, 6]. Both the lack of correct identification of all imaging features and inconsistent interpretation of the SINS score might contribute to this [6]. Recent advances in large language models (LLMs) have demonstrated their potential across a range of radiological applications. Studies have shown that LLMs can assist in aligning clinical decisions with established guidelines, such as the ACR Appropriateness Criteria, sometimes even outperforming radiologists in consistency and accuracy [7]. Other research has explored the utility of LLMs in simplifying radiology reports for patients and non-specialist providers [8], assigning standardized reporting categories like BI-RADS [9], and responding accurately to common cancer-related clinical queries [10]. Moreover, LLMs have shown promise in extracting structured data from free-text radiology reports [11] and answering board-style exam questions with performance comparable to or exceeding that of proprietary models [12]. These findings underscore the growing role of LLMs in enhancing the interpretation, communication, and standardization of radiological information.

Despite their potential, artificial intelligence (AI)-based large language models available to a broad user base like ChatGPT by OpenAI, “Le Chat” by Mistral AI, and Claude by Anthropic are prone to hallucinations and may not correctly apply complex scoring or evaluation systems. Performance and trust of the underlying LLM can be increased through various techniques such as precision prompting, providing task-specific context, or enhancing certainty by a chain-of-thought [13, 14].

In this study, we compared the correctness of SINS classification between human experts, generic state-of-the-art large language models, and models tailored for this specific task through in-context learning. We hypothesized that without refinement, generic large language models would not be capable of correctly applying a classification system that required internal summation of individual items, making multiple decisions for the classification, and accurately counting up results. We anticipated that in-context learning combined with chain-of-thought could significantly improve their performance on these complex, multi-step classification tasks.

## Materials and methods

### Study design and data generation

**Fig. 1:**
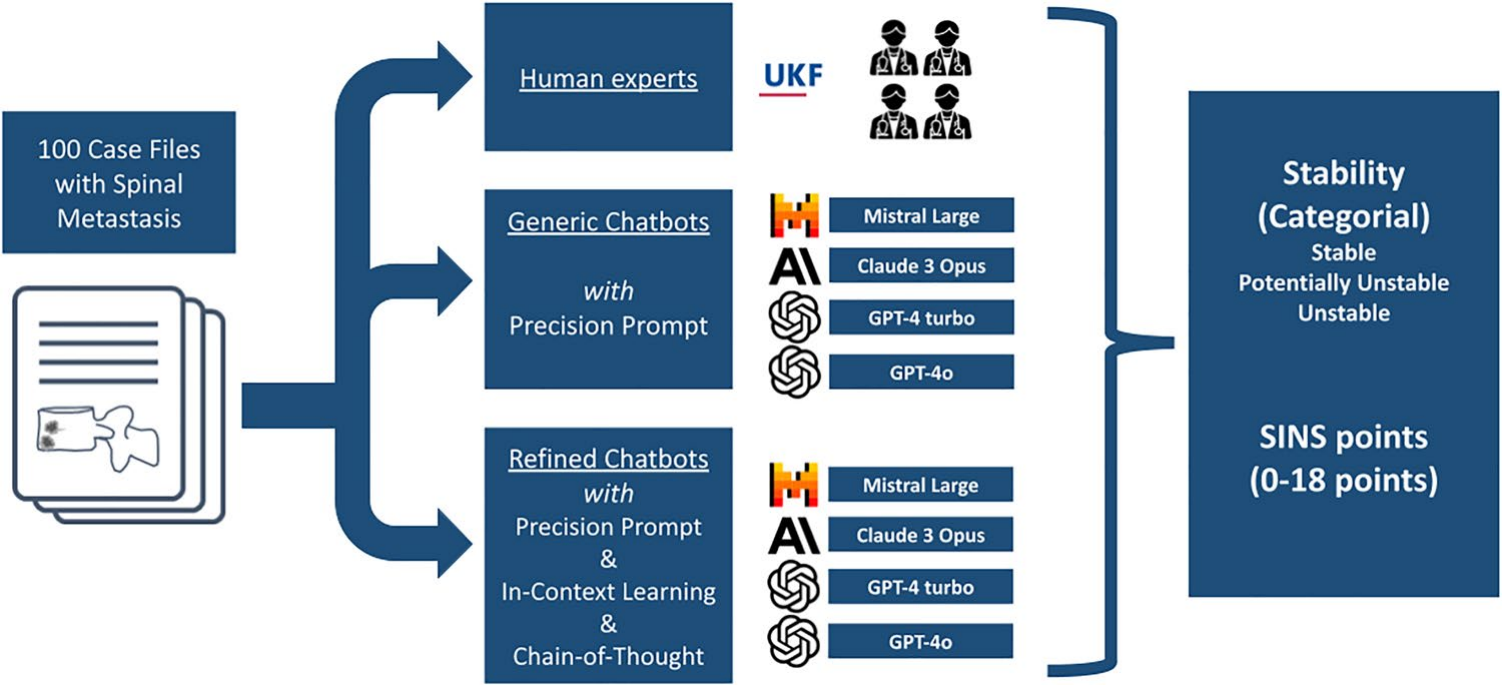
Overview of the study workflow

This proof-of-concept study compared the performance of LLMs with human experts in assessing spinal instability using the SINS. An overview is provided in Fig. 1. A dataset of 100 synthetic radiological reports for CT or MRI, including clinical information and patient history required for SINS assessment, was created by a board-certified radiologist not involved in the subsequent reading. This dataset encompassed a wide range of SINS subscores randomly allocated prior to report generation (mean 8.7 ± 3.1 SINS points (range 1–16) resulting in 24% stable, 67% potentially unstable, and 9% unstable). Upon creation of the reports, we assured that information on all relevant SINS items (i.e., location, pain, appearance of the bone lesion, spinal alignment, vertebral body collapse, and involvement of the posterior spinal elements) was present. The reference standard SINS scores were established by another board-certified radiologist with 13 years of experience and subspecialty in musculoskeletal imaging, strictly following the original SINS definitions and stability cut-offs. These scores served as the gold standard for accuracy and point-offset analyses. As this data was fictitious, institutional review board approval was not required. Also, human ethics and consent to participate declarations is not applicable.

### Assessment of human expert performance

Four board-certified specialists who utilize the SINS score frequently in daily routine (two radiologists with 8 and 7 years of experience and two neurosurgeons with 13 and 11 years of experience) independently evaluated the 100 synthetic reports. They were allowed unlimited time and access to the SINS classification system if needed, mimicking real-world clinical conditions. For each case, the experts provided both the SINS category (0–6 points—stable, 7–12 points—potentially unstable, or 13–18 points—unstable) and the numerical score.

### Assessment of large language model performance

For this study, we selected four state-of-the-art AI-based large language models to test their performance in SINS classification: Mistral Large (released 26.02.2024), Claude 3 Opus (released 04.03.2024), GPT-4 Turbo (released 09.04.2024), and GPT-4o (13.05.2024). On May 27, 2024, each model was tested in two conditions on the 100 radiological reports created:

1. Generic large language model: The case description was provided with a basic precision prompt requesting SINS classification.
2. In-context learning and chain-of-thought: A refined precision prompt was used, providing task-specific context, explanation of the SINS classification system, and guiding the models through a chain-of-thought method.

For the generic large language models, a single case file was provided in a separate session as part of a not further tailored prompt, and the direct output was captured as the response:

*Given the following patient description, please evaluate and provide the overall Spinal instability neoplastic score (SINS) and category:*

*Description:*

*{description}*

TEMPLATE FOR ANSWER:

*Please provide the ANSWER only in a JSON format as follows:*

{{

*"SCORE": "*<*Spinal instability neoplastic score (SINS) value as number from 0 to 18*>*",*

*"CATEGORY": "*<*stable/potentially unstable/unstable*>*"*

}}

Task-specific refinement was analogous for all four large language models and implemented using a precision prompt that defined the task, followed by an explanation of the SINS classification (i.e., in-context learning), and guided the models through a chain-of-thought method. The latter comprised explanation fields for intermediate outputs prior to the final response including the SINS components (i.e., location, pain, bone lesion, alignment, vertebral body collapse/involvement, posterior element involvement) and category, and included a complete explanation of all items required for SINS calculation and classification. The models’ outputs were structured as fixed JavaScript Object Notation (JSON) responses, including scores and explanations for each of the six SINS components and an overall stability classification. Calculation of total points and stability class was done automatically. Each case was processed in a separate session to avoid memory carry-over, and outputs were analyzed without manual correction.

*"Given the following patient description, please evaluate and assign a class and score for each category of the Spinal Instability Neoplastic Score (SINS). For each category, provide a brief explanation for the assigned score.*

*Patient Description:*

{CASE DESCRIPTION}

*Categories and required evaluations are:*

*Location:*

*Occiput-C2: 3 points*

*C3–C6: 2 points*

*[... other location criteria ...]*

*Provide: "vertebra"*

*Provide: "location_class" and "location_score" with explanation as "location_explanation".*

*[... other SINS categories with similar structure ...]*

*Overall SINS:*

*Calculate the total value as sum of all score Values*

*Determine the stability category:*

*total_value* <= *6 "Stable"*

*7* <= *total_value* <= *12: "Potentially unstable"*

*total_value* >= *13 "unstable"*

*Provide: total_calc_explanation, total_value and stability_category*

*Please provide the scores and explanations in a JSON format as follows:*

{

*"location_explanation": "*<*score class explanation*>*",*

*"location_class": "*<*score class name*>*",*

*"vertebra": "*<*Position of the vertebra e.g. T1*>*",*

*"location_score":* <*score value*>*,*

*[... other fields ...]*

*"total_calc_explanation": "*<*total value calculation explanation*>*",*

*"total_value":* <*total value*>*,*

*"stability_category": "*<*stability category*>*"*

}"

### Statistical analysis

The assumption of normal data distribution was assessed with Shapiro–Wilk testing. Inter-rater reliability among human experts was evaluated using quadratic weighted Kappa (QWK). Accuracies in SINS category and points assignment were calculated for both humans and large language models. Here, SINS categorical performance was assessed using the proportion of category accuracy and QWK versus the ground-truth. SINS point allocation was investigated using the exact-match rate for total SINS points, the mean absolute error (MAE), and median absolute offset [IQR]. Human performance was reported per reader and by the median of all raters. Pairwise comparisons were conducted using McNemar’s test with Bonferroni correction applied to the p‑values to account for multiple testing. The absolute offsets of the SINS points were compared using the Kruskal–Wallis test with pairwise comparisons using Dunn’s post-hoc test with Bonferroni adjustment in case of significance. All statistical analyses used Python SciPy 1.11.3, with data presented as median and interquartile ranges or mean and standard deviation as appropriate for continuous variables and as absolute frequencies and percentages for categorical variables. An *α*-level of 0.05 was considered statistically significant.

## Results

### Human performance in SINS classification

**Fig. 2:**
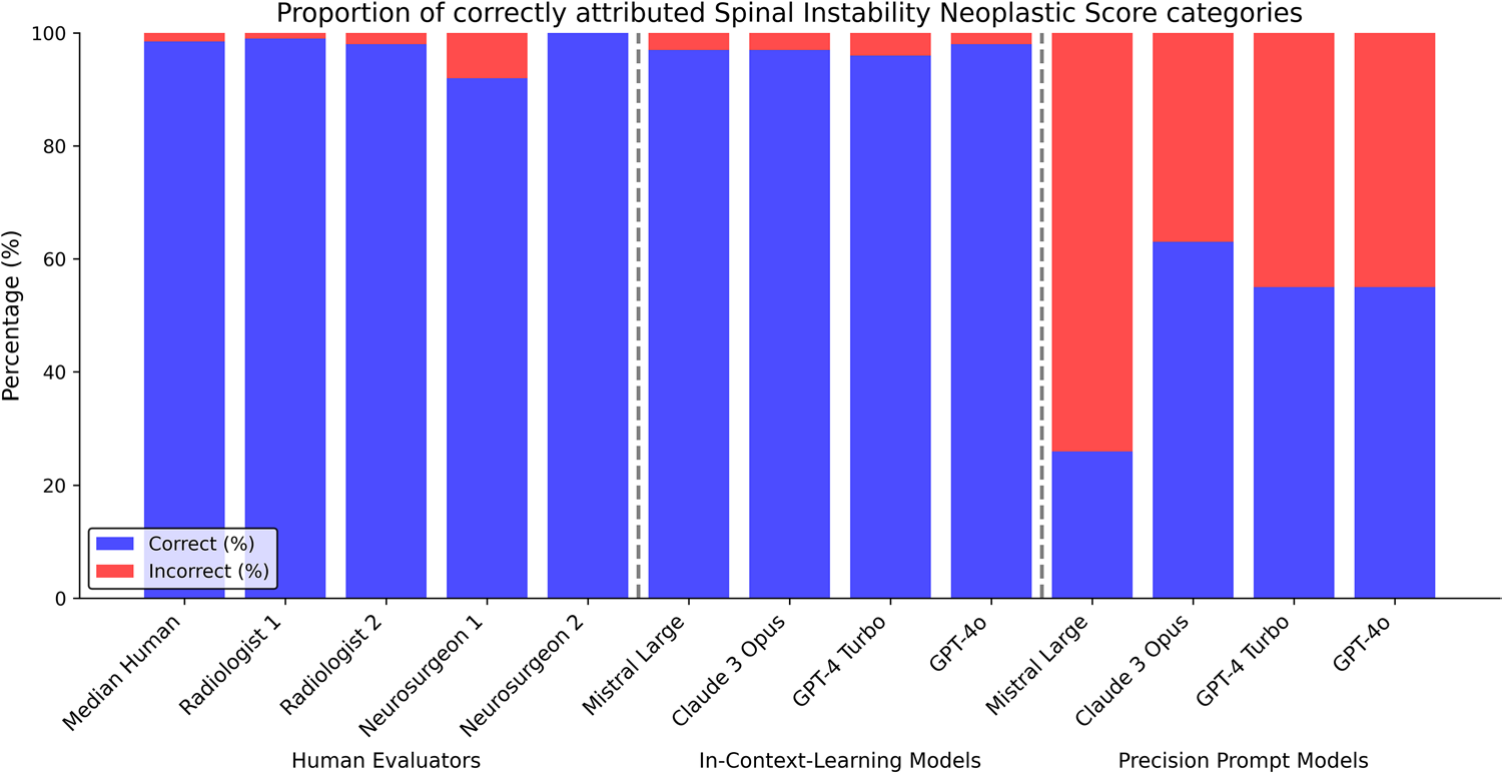
Proportion of correctly attributed Spinal Instability Neoplastic Score categories of human experts, generic chatbots, and chatbots enhanced with in-context learning

**Fig. 3:**
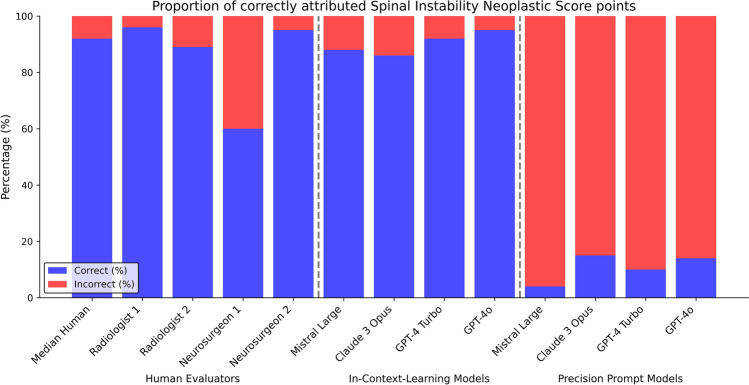
Proportion of correctly attributed Spinal Instability Neoplastic Score points of human experts, generic chatbots, and chatbots enhanced with in-context learning

Human experts demonstrated high performance in SINS classification, with a median of 98.5/100 (98.5%) correct category assignments. The proportion of correctly calculated SINS points was also high, with a median of 92/100 (92%) correct answers and a median score offset of 0 [0–0] across all raters. Inter-rater reliability was almost perfect for categorical assessment (QWK = 0.98) and a low MAE for score values of 0.10. Please see Table 1, Figs. 2 and 3.

**Table 1:** Proportion of correctly identified SINS categories and points

		SINS category correct % (95% CI)	Quadrativ weighted kappa versus ground-truth	SINS points correct % (95% CI)	Median offset [interquartile range]	Mean absolute error
Median of human experts		98.5 (95.4–99.9)	0.976	92 (87.5–97.2)	0 [0–0]	0.10
Generic chatbots	Mistral large	26 (18.4–35.4)	0.175	4 (1.6–9.8)	3 [2–5]	3.50
	Claude 3 Opus	63 (53.2–71.8)	0.455	15 (9.3–23.3)	2 [1–3]	2.22
	GPT-4 Turbo	55 (45.2–64.4)	0.296	10 (5.5–17.4)	2 [1–3]	2.12
	GTP-4o	55 (45.2–64.4)	0.441	14 (8.5–22.1)	2 [1–3]	2.25
Task-specific refinement	Mistral large	97 (91.5–99.0)	0.953	88 (80.2–93.0)	0 [0–0]	0.20
	Claude 3 Opus	97 (91.5–99.0)	0.951	86 (77.9–91.5)	0 [0–0]	0.17
	GPT-4 Turbo	96 (90.2–98.4)	0.935	92 (85.0–95.9)	0 [0–0]	0.11
	GTP-4o	98 (93.0–99.4)	0.967	95 (88.8–97.8)	0 [0–0]	0.07

### Generic large language models in SINS classification

Generic large language models without task-specific refinement performed poorly in allocation of the stability category, achieving 63.0% (Claude 3 Opus), 55.0% (GPT-4 Turbo and GPT-4o), and 26.0% (Mistral Large) correct classifications. QWK in comparison with the ground-truth ranged between 0.18 and 0.46. SINS points calculation ranged between 15% (Claude 3 Opus), 14% (GPT-4o), 10% (GPT-4 Turbo) and 4% (Mistral Large) correct scores. The median offset from the correct SINS points was high for all generic large language models: Mistral Large 3 [2–5], Claude 3 Opus 2 [1–3], GPT-4 Turbo 2 [1–3], and GPT-4o 2 [1–3]. MAE was between 2.13 and 3.50. We did not find significant differences in performance across the generic large language models neither for the SINS category nor points (*p* > 0.12), though they were all outperformed by human expert readers regarding both SINS category and points (*p* < 0.001). An in-depth analysis of cases with false SINS attribution revealed that most errors were positive offsets, indicating systematic overestimation of instability. This occurred especially in cases that included trigger words such as collapse, kyphotic deformity, bilateral posterior elements, extensive/advanced structural impact, mechanical pain wording (worse with load or movement). Associated negations or qualifiers that should downscore SINS components (i.e., alignment intact, no posterior involvement) were often ignored.

Additionally, models falsely attributed higher SINS points if the reports included mentions of collapse without clear differentiation between < 50% versus > 50% vertebral height loss but rather wordings like “approximately one third”.

### Large language models enhanced with in-context learning

Refinement of the large language models via in-context learning significantly improved their performance compared with their generic counterpart (all pairwise comparisons *p* < 0.001 for both the SINS category and points) with correct classification rates ranging from 96.0% to 98.0% (GPT-4o: 98.0%, Mistral Large and Claude 3 Opus: 97.0%, and GPT-4 Turbo: 96.0%). This was corroborated by high QWK upon comparison with the ground truth (0.94–0.97). GPT-4o and GPT-4 Turbo performed best by providing the correct SINS points in 95 and 92 of the 100 cases, whereas Mistral Large and Claude 3 Opus provided 88 and 86 correct points, respectively. All large language models did not perform significantly different compared with the median of human expert readers (*p* > 0.99), and the median offset of the score was comparable with 0 [0–0] for all large language models with in-context learning, as was the MAE that ranged between 0.07 and 0.20. An exemplary case file and responses of the generic and task-specifically refined Mistral Large are provided in the Supplement.

## Discussion

In this comparative study, task-specific refinement of AI-based large language models via in-context learning significantly improved the correct classification of vertebral stability of state-of-the-art large language models reaching human expert level.

The poor performance of generic large language models underscores the risks of integrating unrefined LLMs into clinical decision-making. Without contextual information and guidance via precision prompting and chain-of-thought, these state-of-the-art LLMs failed to consistently apply the SINS criteria, potentially leading to dangerous misclassifications if used in practice [4, 14].

Though recent studies on the applicability of generic LLM into various tasks in patient management provided encouraging results, our findings in one of the first studies that comprehensively compared the performance of multiple publicly available state-of-the-art LLMs indicate that their generic versions should only be used with great caution in a clinical context and cannot reliably cope with the broad range of the field of radiology but rather require task-specific refinement [7, 15]. In detail, refinement with task-specific context through our in-context learning approach significantly improved performance, reaching true positive rates of 96–98%, i.e., an improvement of 71–84% more correctly allocated SINS points. Noteworthy, all investigated LLMs benefitted from this reaching comparable performance. This remarkable improvement aligns with recent advancements in LLM capabilities [9–11, 16], as, e.g., demonstrated by Brown et al. in their work on few-shot learning [17]. In general, strategies in prompt-engineering substantially improve large language models’ responses [13]. A framework to provide task-specific context, such as the one we employed, streamlines the large language models’ response generation, thus reduces the risk for hallucinations and therefore enhancing trust in the output [18]. Moreover, the employed chain-of-thought allows for an insight into the large language models’ decision-making further enhancing trust and potentially refining incorrect responses as demonstrated by the responses in the Supplement. Moreover, in-context learning has to be considered a powerful approach to provide task-specific knowledge to a LLM as in contrast to, e.g., vectorized knowledge [7, 18] it is easily applicable in the front-end environment of publicly available large language models, too. This allows for a rapid adaptation of the task-specific knowledge base and facilitates integration in current clinical routine.

Most previous studies in the medical field on LLM performance in medical classification tasks investigated binarized problems or simple classification systems. This reached from binary classifications such as identifying an ankle fracture from a radiological report [19], to grading of prostate, breast or hepatic cancer using MRI reports according to the LI-RADS, BI-RADS or PI-RADS [9, 20, 21] and tumor staging of lung cancer [22]. In general, generic LLMs were capable of coping with simple classification systems but failed more complex ones such as the different RADS or lung cancer TNM.

In contrast, we investigated a complex scoring system that requires the capability of summing individual subscores to a final score and subsequently attributing a category (i.e., stable, potentially unstable, and unstable) [4].

Whereas most studies on the applicability of LLMs in radiological tasks investigated either one LLM [19, 22] or compared either various versions of GPT by OpenAI [7, 18, 23, 24] or various different LLMs in their generic versions [9, 20], we herewith present a comprehensive evaluation of both the generic and task-specifically refined versions of four state-of-the-art LLMs. This allowed for revealing insufficient performance in the application of the SINS classification system for all generic large language model versions despite a precision prompt but a substantial improvement with noteworthy performance for all large language models upon further refinement.

The proposed framework might be employed as a tool to provide SINS scores for clinical decision-making, second reader for quality assurance, or to retrospectively assign SINS scores to existing reports with this information lacking, though only if the necessary information for scoring was provided. In clinical routine, this could be employed to automatically calculate the SINS from a free-text report. This would allow the radiologist to focus on the image interpretation and outsource the subsequent scoring to increase workflow efficiency. A further refined large language models could additionally highlight if information for appropriate SINS classification is lacking, e.g., if the involvement of posterior elements was not reported. This would increase quality of reporting in the presence of spinal metastatic disease and thus increase patient safety. Alternatively, the proposed model could be employed in parallel to human SINS classification to reduce human error, especially in vague cases. Such an application would be of especial worth in training of early career radiologists who are not yet sufficiently familiar with the SINS by facilitating the comparison of human and model-based scorings. Lastly, the LLM refined in this study could be retrospectively applied on radiological reports to either compare the output with human rating for quality management or improvement, or in case of no previous usage of the SINS to allocate SINS scores or categories to imaging data. The latter approach would allow for quality management, too, and could be compared with treatment courses of patients to identify structural concerns in clinical workflows.

Furthermore, the storage of large language model responses in a JSON format allows for the automated integration into a Fast Healthcare Interoperability Resources (FHIR) environment enabling structured output for patient-specific precision medicine and easily assessing individual items of a complex system. Additionally, the application on existing data for quality purposes and research is feasible [11, 25].

The use of synthetic reports, while allowing for controlled evaluation, may not fully capture the nuances and complexities of real clinical cases and thus reduce generalizability. The employed approach with synthetic reports encompassing a broad range of possible SINS scores and combinations enabled a comprehensive comparison between human expert performance and large language model capabilities in SINS assessment. The use of synthetic data was necessary in this initial proof-of-concept study to systematically assess LLM performance in a controlled environment (e.g., by ensuring the dataset encompasses a sufficient spread of SINS points), allowing for targeted testing of the task-specific refinement method in a broad range of possible cases. However, synthetic reports do not reflect the ambiguities, incompleteness, and linguistic variability that characterize clinical documentation. Real clinical reports often contain diverse phrasing, implied or missing information, institutional style differences, and occasional reporting errors, all of which pose substantial challenges for automated interpretation by LLMs. Here, the involvement of more radiologists—preferably from various centers—in the creation of the reports would have increased linguistic variability and should be included in future research. The observed inter-rater reliability was higher than in previous studies [5, 6, 26], which we attribute to the ability to reference the SINS classification system and the use of standardized reports rather than direct image interpretation. This design was chosen to resemble clinical routine. However, the focus on textual reports rather than direct image analysis diverges from typical radiological practice, and future studies should explore multimodal AI approaches combining text and image analysis as the latter is feasible with AI, too [27, 28]. Exploration of multimodal AI approaches, combining text-based models with image analysis, could provide a more comprehensive tool for radiological assessment, as suggested by Huang et al. [29]. Future research should investigate real-world data, which was not feasible in our study due to data protection regulations. Transitioning the proposed approach to clinical settings will require extensive validation on large, diverse datasets of real radiology reports. This includes addressing challenges such as heterogeneous language use, underreporting of relevant findings, and incomplete documentation, all of which may hinder accurate score allocation, a challenge that human readers have to face as well. Upon using real data from clinical routine, LLMs might also be prompted to indicate lacking information and thus not scorable reports.

In conclusion, task-specifically refined large language models with in-context learning and a chain-of-thought approach reached human expert-level performance in the application of the SINS scoring system, while generic versions of the same models misclassified a great proportion of cases with potentially great consequences.

## Data Availability

Data generated or analyzed during the study are available from the corresponding author by request.
